# Supplementary material for: Effects of Molecular Iodine/Chemotherapy in the Immune Component of Breast Cancer Tumoral Microenvironment
Source: Biomolecules. 2021 Oct 12;11(10):1501. doi: 10.3390/biom11101501 (PMC8533888; doi:10.3390/biom11101501)
Supplement: Supplementary file 1 [file biomolecules-11-01501-s001.zip › biomolecules-1394051-supplementary.pdf]

Table S1: Primers for RT-PCR genes expression

GENES	REFERENCE	OLIGOS (FWD/RVS)	SIZE (BP)
**IL-12RB**	NM_001290023.2	CCCAGCAGCCCATGGA	92
		TCCTGGGCCACATTCATCTC	
**T-BET**	NM_013351.2	GCTGCCCCACTTTGAACATCAG	164
		GGAGGCTTGGGGGAGAGAGAAT	
**IFNγ**	NM_000619.3	GGCAAGGCTATGTGATTACAAGG	96
		CATCAAGTGAAATAAACACACAACCC	
**TGFβ**	NM_00660.7	ATCGGTGCTGACGCCTGGCCC	197
		TGCTGTTGTACAGGGCGAGCA	
**GATA-3**	NM_002051.3	ACGTCTCACTCTCGAGGCAGCATG	564
		GAAGTCCTCCAGCGCGTCATGCAC	

Table S2: Primers for Methylated promoter regions (After Bisulfite conversion) and for housekeeping gene MLH-1 also for after bisulfite conversion.

GENES	REFERENCE	OLIGOS (FWD/RVS)	SIZE (BP)
**IFNγ**	Methprimer	TTTTGATTAATATAGTGAAATTTCGT	194
		TCACCCAAACTAAAATACAATAACG	
**TGFβ**		GTTTTTTTGTGTTTTATTTTTCGG	142
		AAACAAAATACTACCTCCTAACGAC	
**MLH-1**	GenBank Accession #: U83845	GGAGTGAAGGAGGTTACGGGTAATG	182
		AAAAACGATAAAACCCTATACCTAATCTATC	

Table S3: Primers for Unmethylated promoter regions (After Bisulfite conversion)

GENES	REFERENCE	OLIGOS (FWD/RVS)	SIZE (BP)
**IFNγ**	Methprimer	TTGATTAATATAGTGAAATTTTGT	190
		ACCCAAACTAAAATACAATAACACA	
**TGFβ**		TTTTTTTGTGTTTTATTTTTTGG	141
		AAACAAAATACTACCTCCTAACAAC	
